# Frequency-Specific Regional Homogeneity Alterations in Tourette Syndrome

**DOI:** 10.3389/fpsyt.2020.543049

**Published:** 2020-12-17

**Authors:** Yu-Ting Lou, Xiao-Long Li, Ye Wang, Gong-Jun Ji, Yu-Feng Zang, Jue Wang, Jian-Hua Feng

**Affiliations:** ^1^Department of Pediatrics, The Second Affiliated Hospital, School of Medicine, Zhejiang University, Hangzhou, China; ^2^Institutes of Psychological Sciences, Hangzhou Normal University, Hangzhou, China; ^3^Zhejiang Key Laboratory for Research in Assessment of Cognitive Impairments, Hangzhou, China; ^4^Center for Cognition and Brain Disorders and the Affiliated Hospital, Hangzhou Normal University, Hangzhou, China; ^5^Department of Medical Psychology, Chaohu Clinical Medical College, Anhui Medical University, Hefei, China; ^6^Collaborative Innovation Centre of Neuropsychiatric Disorder and Mental Health, Anhui Province, Hefei, China; ^7^Institute of Sports Medicine and Health, Chengdu Sport University, Chengdu, China

**Keywords:** Tourette syndrome, resting-state functional MRI, regional homogeneity, frequency-specific, network

## Abstract

Tourette syndrome (TS) is a developmental neuropsychiatric disorder with onset during childhood. Because of its complex spectrum of phenotypes, the underlying pathophysiology of TS is still unclear. Resting-state functional magnetic resonance imaging demonstrated aberrant spontaneous neural synchronization in conventional frequency band (0.01–0.08 Hz) in TS. No published studies have reported abnormalities of local synchronization across different frequency bands. We estimated the alterations of local synchronization across five bands ranging from 0 to 0.25 Hz. Seventy-nine children with TS and 63 age-, sex-, and handedness-matched healthy children were recruited. Frequency-specific regional homogeneity (ReHo) and independent component analysis were used to identify functional alterations between TS and healthy children. TS patients showed significantly increased ReHo in the left precentral gyrus and decreased ReHo in the right operculum. Abnormal ReHo alterations of the superior frontal gyrus, superior parietal gyrus, anterior cingulate gyrus, putamen, superior temporal gyrus, and operculum were observed in different frequency bands. TS patients showed increased connectivity of the right superior frontal gyrus within the left executive control network. In addition, a significantly negative correlation was found between Yale Global Tic Severity Scale (YGTSS) vocal score and ReHo values of the right operculum in the highest frequency bands (0.198–0.25 Hz), while a significant positive correlation was found between YGTSS motor score and altered connectivity of the right superior frontal gyrus. The present study revealed frequency-specific abnormal alterations of ReHo in the whole brain and altered connectivity within the executive control network of TS children. Its neural importance and clinical practicability require further investigation.

## Introduction

Tourette syndrome (TS) is a developmental neuropsychiatric disorder with an onset during childhood, defined by the co-occurrence of motor and phonic tics lasting at least 12 months. Tics typically show a waxing and waning pattern of severity, intensity, and frequency (1). The prevalence of TS for children in China is 1.7% (2). Previous studies showed that ~90% of TS children have comorbid conditions, including obsessive–compulsive disorder (OCD), attention-deficit hyperactivity disorder (ADHD), rage attacks, sleep disturbances, and depressive symptoms (3), which might have a negative impact on their quality of life (4). Moreover, the underlying pathophysiology of TS is controversial and poorly understood because of the complex spectrum of phenotypes involved in TS (5).

Advanced neuroimaging methods have been used to investigate the neural basis of TS over the last few decades. Many neuroimaging studies using structural magnetic resonance imaging (MRI), positron emission tomography (PET), and diffusion tensor imaging techniques have reported abnormalities in the basal ganglia and associated thalamic and frontal cortical regions (6), supporting a hypothesis of dysfunction in the cortico-striato-thalamo-cortical (CSTC) networks in TS (7). A recent meta-analysis of task-based neuroimaging studies showed that TS patients had abnormal activation in the prefrontal, anterior cingulate, motor preparation cortices, and sensory and temporo-parietal association cortices (8). A previous study using functional MRI (fMRI) showed abnormal functional connections in the executive control network, especially in the fronto-parietal network in TS patients (9). This suggested that the broadly distributed involvement of multiple cortical regions or brain networks, in addition to the CSTC network, might play a critical role in TS pathophysiology.

Because TS children cannot perform specified tasks well, there have been few task-related fMRI studies in TS children. Resting-state fMRI (RS-fMRI) has the advantage of measuring spontaneous neural activity such as regional homogeneity (ReHo) in the TS population (10, 11). A previous study of TS with a small sample size (*n* = 21) showed decreased ReHo in the right cerebellum that was positively correlated with disease duration (11); however, no cortical regions were identified with abnormal ReHo. That study only observed the local synchronization of spontaneous RS-fMRI signals at a low frequency band (0.01–0.08 Hz), and therefore information about other lower- or higher-frequency realms is unknown (12). In contrast, a broader band of 0.01–0.08 Hz, containing several mixed physiological alterations of potentially specific frequencies, may lead to negative or inexact results. Zuo et al. discovered that even in the conventional low-frequency band (<0.1 Hz), blood oxygenation level-dependent (BOLD) fluctuations were stronger at 0.01–0.027 Hz in the subcortical region and at 0.027–0.073 Hz in the cortical region (13). Frequency characteristic analysis demonstrated that ReHo oscillations lower than 0.02 Hz mainly occurred in the putamen and that higher-frequency oscillations mainly occurred in limbic areas (>0.08 Hz) (14). Thus, a frequency-specific approach may provide more information than that of the conventional band (<0.1 Hz) to help understand the local BOLD activity of the cortical regions in TS.

To discover frequency-specific functional alterations that might have an important role in the pathophysiology of TS, we used RS-fMRI in a frequency-specific manner (0–0.01 Hz, 0.01–0.027 Hz, 0.027–0.073 Hz, 0.073–0.198 Hz, and 0.198–0.25 Hz) to compare TS patients with healthy controls (HC) (13, 15). Independent component analysis (ICA) was used to detect connectivity differences within brain networks between TS and HC groups. In the current study, we included subjects with comorbid symptoms who are most typical of TS [as “pure” TS without any comorbidity occurs in only 10% of patients (3)] to obtain generalizable results for clinical practice.

## Materials and Methods

### Participants

Ninety-four children with TS were recruited from outpatient clinics of the Second Affiliated Hospital, Zhejiang University School of Medicine. All patients met the Diagnostic and Statistical Manual of Mental Disorders, 4th edition, text revision (DSM-IV-TR) criteria. Sixty-three age-, sex-, and handedness-matched healthy children without any neurological or psychiatric disorders were recruited as HC. Participants were excluded if they had (i) any neurological (other than tics) or psychiatric diseases (other than ADHD and OCD), (ii) structural abnormalities on visual inspection of structural imaging, and (iii) head motion exceeding 3 mm in translation or 3° rotation in any direction. After head-motion control, fifteen patients were excluded. Seventy-nine TS patients were eligible to take part in the current study. All clinical evaluations were performed on the day of acquisition of MRI scans by an experienced pediatric neurologist (Table 1). The Yale Global Tic Severity Scale (YGTSS) was used to assess current tic severity. The Swanson, Nolan, and Pelham IV Scale (SNAP-IV) was used to assess ADHD symptoms, and the Children's Yale–Brown Obsessive Compulsive Scale (CY-BOCS) was used to quantify OCD symptoms. Twenty-five children with TS in this study were using psychoactive medication during the MRI scan. Eighteen children with TS were on monotherapy (Tiapride 11/18, Topamax 5/18, Haloperidol 2/18), while seven subjects were taking combination drug therapy. Informed written consent from parents and assent by children for TS and HC were obtained before participation. The study was approved by the Medical Ethics Committee of the Center for Cognition and Brain Disorders, Hangzhou Normal University, China.

**Table 1:** Demographic variables and clinical characteristics.

**Characteristics**	**TS *(n* = 79)**	**HC (*n* = 63)**	***P*-value**
Age (years)	9.56 ± 2.50	9.35 ± 2.19	0.599
Sex, male/female	74/5	53/10	0.066
Handedness, right/left	78/1	63/0	0.370
Duration (years)	2.28 ± 2.14	–	–
YGTSS (total score)	22.00 ± 7.58	–	–
YGTSS (motor score)	13.32 ± 3.49	–	–
YGTSS (vocal score)	8.67 ± 5.94	–	–
SNAP-IV	12.86 ± 7.64	–	–
CY-BOCS	0.52 ± 2.39	–	–
Drug information			
Tiapride	11	–	–
Topamax	5	–	–
Haloperidol	2	–	–
Combination drugs	2	–	–

*Date are expressed as mean ± SD. YGTSS, Yale Global Tic Severity Scale; SNAP-IV, Swanson, Nolan, and Pelham IV Scale; CY-BOCS, Children's Yale–Brown Obsessive Compulsive Scale*.

### Image Acquisition

MRI images were acquired on a 3.0-Tesla MRI scanner (GE Discovery 750 MRI, General Electric, Milwaukee, WI, USA) at the Center for Cognition and Brain Disorders, Hangzhou Normal University. Foam pads were used to minimize head motion for all subjects. A gradient-recalled echo planar imaging sequence (repetition time = 2,000 ms, echo time = 30 ms, and flip angle = 90°) was used to obtain functional images. Forty-three axial slices (field of view = 220 × 220 mm, matrix = 64 × 64, slice thickness/gap = 3.2/0 mm, and 240 volumes) were acquired. Participants were instructed to rest with their eyes closed, not to think of anything in particular, and not to fall asleep. Three-dimensional T1-weighted images were obtained in the sagittal orientation using a magnetization-prepared rapid acquisition gradient-echo sequence (repetition time = 8.068 ms, echo time = 3.136 ms, flip angle = 8°, field of view = 250 × 250 mm, matrix = 256 × 256, slice thickness/gap = 1/0 mm, and 176 slices). After each scanning session, the responsiveness of the subjects was determined by asking whether they had fallen asleep during the scan.

### Image Data Preprocessing

Statistical Parametric Map (SPM12, http://www.fil.ion.ucl.ac.uk/spm) software and the Data Processing & Analysis for Brain Imaging (DPABI) toolbox (http://rfmri.org/dpabi) were used for preprocessing (16). The first 10 image volumes of fMRI scans were discarded for scanner calibration and the subject's adaptation to the scanning noise. After slice time correction, functional images were spatially realigned to the first image of each session for head motion correction. Head movements assessed by the realignment parameters were tolerated up to ±3 mm and ±3°. Then, several nuisance variables including a linear trend, head-motion parameter (the Friston 24-parameter model) (17), white matter, cerebrospinal fluid, and global mean signals were regressed out (16). Individual T1 images were coregistered to functional images, then segmented (gray matter, white matter, and cerebrospinal fluid) and normalized to Montreal Neurological Institute (MNI) space. The transformation matrix obtained from T1 segmentation was applied to the functional images. The normalized functional images were resampled to 3 × 3 × 3 mm. Finally, we performed bandpass filtering (0.01–0.08 Hz, 0–0.01 Hz, 0.01–0.027 Hz, 0.027–0.073 Hz, 0.073–0.198 Hz, 0.198–0.25 Hz). ReHowas calculated (27 voxels) and ReHo maps from each frequency band for each individual were divided by the global mean ReHo value and then spatially smoothed with a Gaussian kernel (full width at half maximum = 6 mm). Ultimately, smReHo maps were used for statistical analysis.

For ICA, fMRI data were spatially smoothed with 6 mm full width half maximum Gaussian kernel after normalization. Subsequently, Group ICA of the fMRI Toolbox v3.0b (GIFT, http://icatb.sourceforge.net) was used to calculate spatially independent components (ICs). Dimension estimation of data from both groups was conducted using the minimum description length (MDL) criterion to determine the number of ICs (18). Then, individual maps in each group were conjoined and the temporal dimension of the convergent data was reduced via principal component analysis, followed by IC estimation. For all subjects, the spatial component maps were converted into z-score maps.

**Figure 1:**
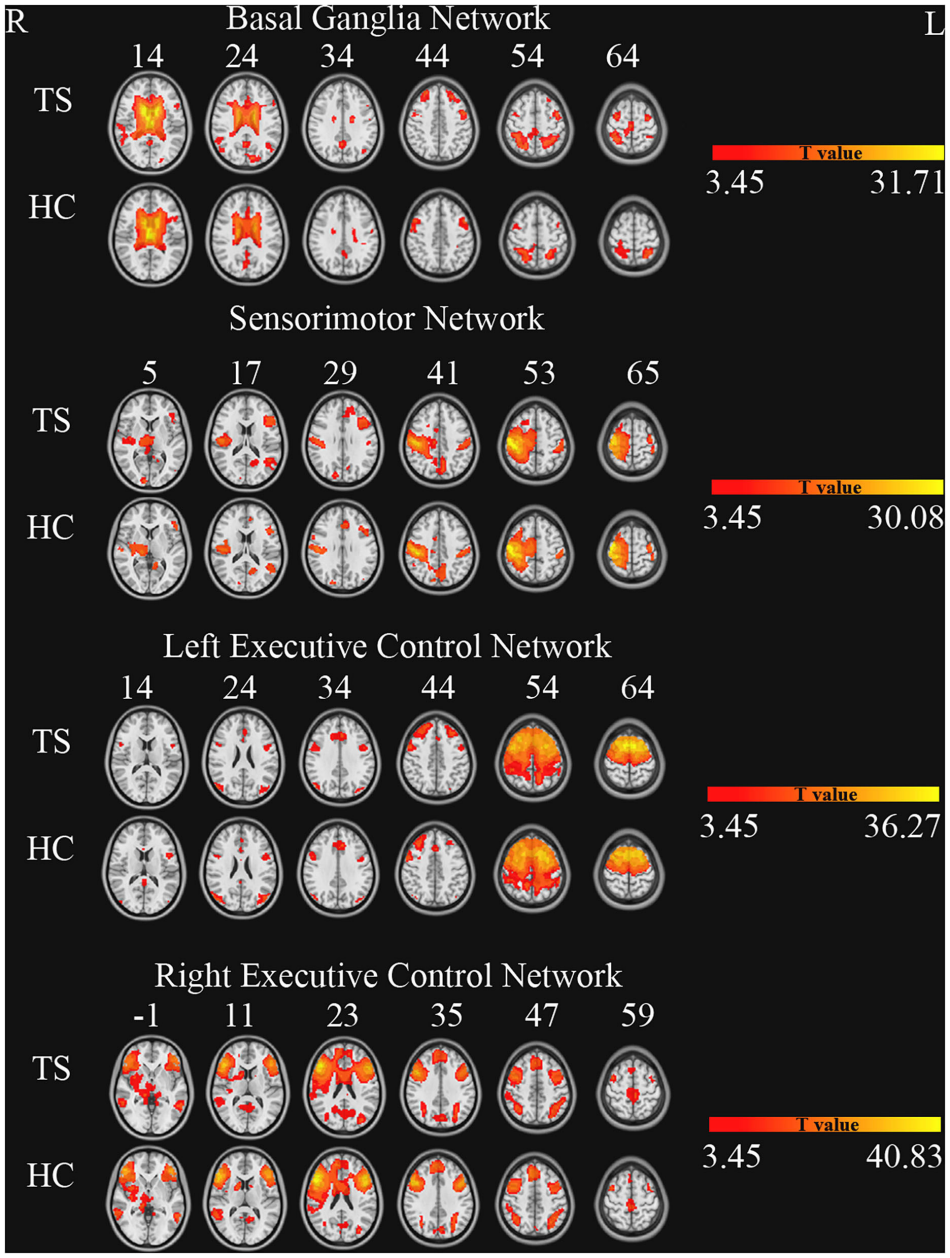
Brain networks of independent component analysis for each group (one sample *t*-test, GRF correction, single voxel *p* < 0.001, cluster level *p* < 0.05).

The number of ICs in the two groups (TS and HC) was 43. These components were selected based on the largest spatial correlation with a specific resting-state network template (http://findlab.stanford.edu/functional_ROIs.html) for further analysis. The brain networks included basal ganglia, sensorimotor, and the left and right executive control networks (one sample *t*-test, multiple-comparison correction based on Gaussian random field theory, single voxel *p* < 0.001, cluster level *p* < 0.05, Figure 1).

### Statistical Analysis

Two-way repeated-measures analysis of variance (ANOVA) was performed to examine the effects of group and frequency band on the ReHo maps. Group (TS and HC) served as a between-subject factor and frequency band (0–0.01 Hz, 0.01–0.027 Hz, 0.027–0.073 Hz, 0.073–0.198 Hz, and 0.198–0.25 Hz) served as a within-subject factor. An F-map of the “frequency by group” interaction effect and an F-map of the group main effect were obtained. Multiple-comparison correction was conducted based on Gaussian random field (GRF) theory (single voxel *p* < 0.001, cluster level *p* < 0.05). The peak ReHo values of the clusters surviving from the group main effect were extracted and then entered SPSS software for two-sample *t*-tests in each frequency band

The peak ReHo values of TS patients were correlated against the clinical measurements, including YGTSS scores and SNAP-IV scores.

To explore connectivity differences between the two groups, two sample *t*-tests were performed within each network. Multiple-comparison correction was conducted based on GRF theory (single voxel *p* < 0.001, cluster level *p* < 0.05).

## Results

There were no significant differences in age (*p* = 0.599), sex (*p* = 0.066), and handedness (*p* = 0.370) between the two groups. The detailed demographic variables and clinical characteristics of participants are shown in Table 1.

### Differences in Regional Homogeneity

**Figure 2:**
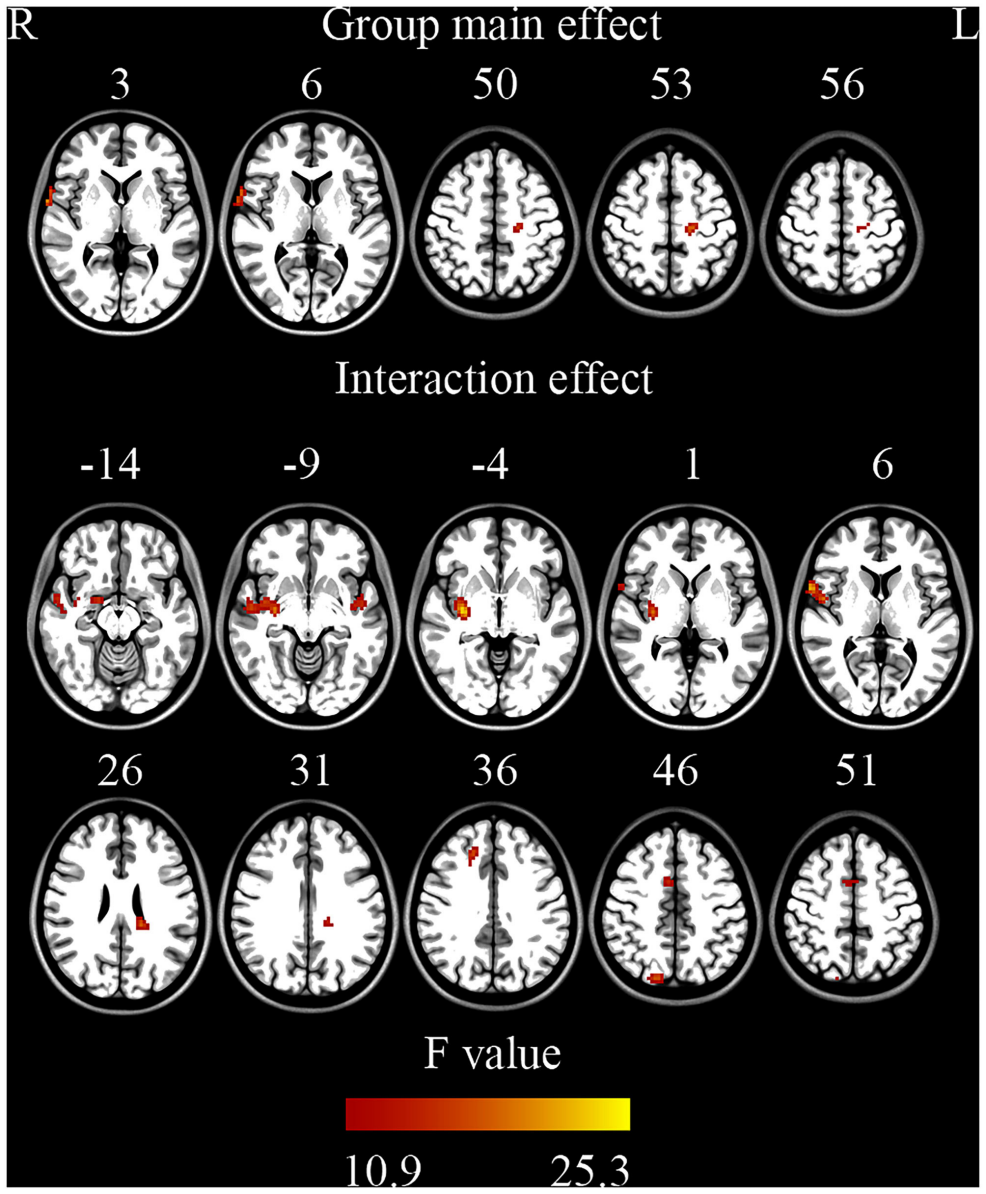
Brain regions showing significant differences on ReHo (ANOVA) (GRF correction, single voxel *p* < 0.001, cluster level *p* < 0.05).

**Figure 3:**
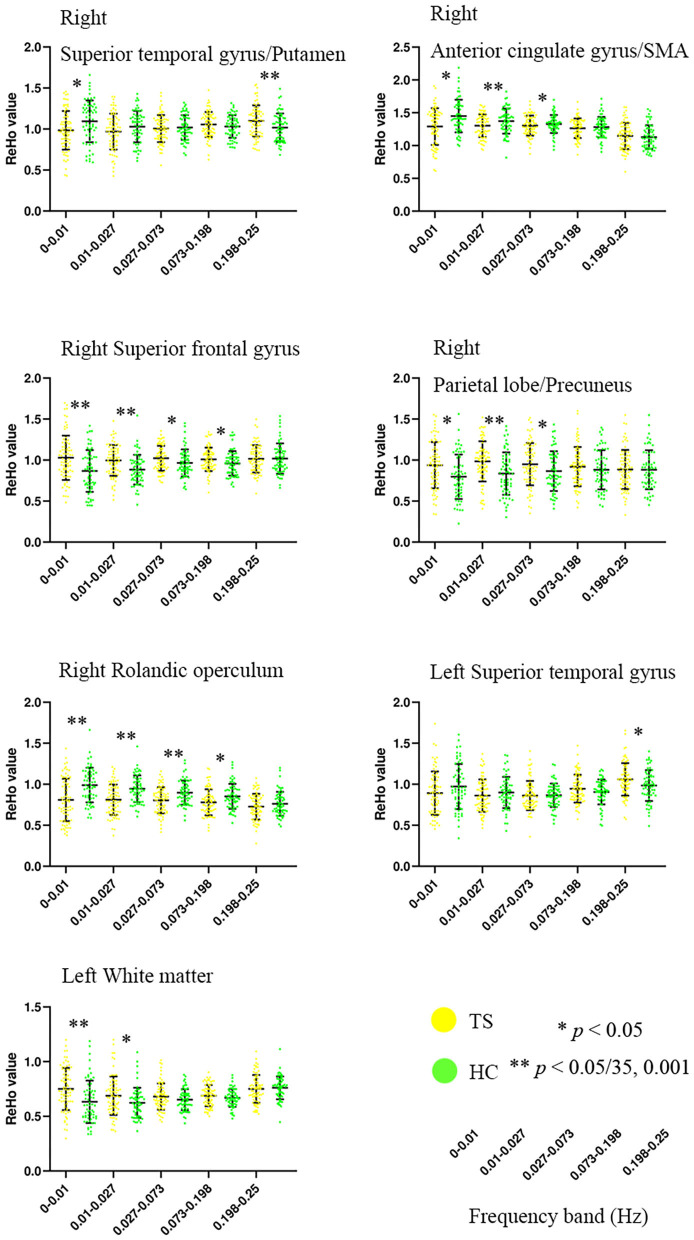
Altered ReHo of brain regions in TS patients across five frequency bands ranging from 0 to 0.25 Hz. SMA, supplementary motor cortex. **Bonferroni correction, i.e., 0.05/35 = 0.001.

ANOVA showed a significant main effect of group and a significant interaction effect (Figure 2, Table 2). For the main effect of group, the brain regions included the left precentral gyrus and the right operculum (frontal and temporal lobes). For the interaction effect, the brain regions included the right anterior cingulate gyrus (ACC)/supplementary motor cortex (SMA), right superior frontal gyrus, right Rolandic operculum, right putamen, right superior parietal gyrus, left superior temporal gyrus, and left white matter. Generally, in TS patients, the right superior frontal, and superior parietal gyrus showed an increased ReHo in lower bands and turned to normal in the higher bands, while the right Rolandic operculum and ACC/SMA showed a decreased ReHo in lower bands and turned to normal in higher bands. The right putamen and the left superior temporal gyrus showed a trend of decreased ReHo in lower frequency bands and increased ReHo in higher frequency bands (Figure 3, Table 3).

**Table 2:** Brain regions showed significant differences (ANOVA).

**Brian regions**	**Hemisphere**	**Brodmann's area**		**MNI coordinate** **(X, Y, Z)**		**Cluster size** **(mm^**3**^)**	***F*-value**	***P*-value**
**Main effect of group**								
Operculum (frontal and temporal lobe)	R	48	66	3	3	621	15.95	<0.05
Precentral gyrus	L	N/A	−18	−21	54	621	14.96	<0.05
**Interaction effect of group** **×** **frequency**								
Superior temporal gyrus/putamen	R	20	30	−12	−6	4,833	25.40	<0.05
Anterior cingulate gyrus/supplementary motor cortex	R	24	6	6	45	783	14.98	<0.05
Superior frontal gyrus	R	32	18	33	36	756	16.78	<0.05
Parietal lobe/precuneus	R	7	15	−81	48	702	17.81	<0.05
Rolandic operculum	R	48	63	9	6	2,511	21.58	<0.05
Superior temporal gyrus	L	48	−48	−6	−9	702	15.10	<0.05
White matter	L	N/A	−18	−33	27	999	17.89	<0.05

*P-value, GRF correction, single voxel p < 0.001, cluster level p < 0.05*.

**Table 3:** Details of simple effect for the brain regions in ANOVA (interaction effect of group × frequency).

**Frequency band**	**TS (*n* = 79) mean ± SD**	**HC (*n* = 63) mean ± SD**	***T*-value**	***P-*value**
**Right superior temporal gyrus/putamen**				
0–0.01 Hz	0.98 ± 0.24	1.09 ± 0.26	2.659	0.0088^*^
0.01–0.027 Hz	0.97 ± 0.22	1.03 ± 0.19	1.728	0.0862
0.027–0.073 Hz	1.01 ± 0.16	1.02 ± 0.15	0.472	0.6377
0.073–0.198 Hz	1.06 ± 0.15	1.03 ± 0.14	1.011	0.3139
0.198–0.25 Hz	1.10 ± 0.19	1.02 ± 0.17	2.681	0.0082^*^
**Right ACC/SMA**				
0–0.01 Hz	1.29 ± 0.28	1.45 ± 0.25	3.547	0.0005^**^
0.01–0.027 Hz	1.30 ± 0.17	1.37 ± 0.19	2.294	0.0233^*^
0.027–0.073 Hz	1.30 ± 0.15	1.33 ± 0.14	1.030	0.3049
0.073–0.198 Hz	1.26 ± 0.15	1.28 ± 0.16	0.645	0.5201
0.198–0.25 Hz	1.15 ± 0.20	1.13 ± 0.18	0.583	0.5609
**Right superior frontal gyrus**				
0–0.01 Hz	1.03 ± 0.27	0.87 ± 0.26	3.601	0.0004^**^
0.01–0.027 Hz	1.00 ± 0.19	0.88 ± 0.18	3.595	0.0004^**^
0.027–0.073 Hz	1.02 ± 0.15	0.97 ± 0.17	2.122	0.0356^*^
0.073–0.198 Hz	1.01 ± 0.14	0.96 ± 0.15	1.995	0.0479^*^
0.198–0.25 Hz	1.02 ± 0.17	1.02 ± 0.19	0.098	0.9224
**Right parietal lobe/precuneus**				
0–0.01 Hz	0.94 ± 0.28	0.80 ± 0.27	2.999	0.0032^*^
0.01–0.027 Hz	0.98 ± 0.24	0.84 ± 0.26	3.504	0.0006^**^
0.027–0.073 Hz	0.95 ± 0.26	0.87 ± 0.24	1.996	0.0479^*^
0.073–0.198 Hz	0.92 ± 0.24	0.88 ± 0.24	0.936	0.3510
0.198–0.25 Hz	0.89 ± 0.24	0.88 ± 0.24	0.072	0.9430
**Right Rolandic operculum**				
0–0.01 Hz	0.81 ± 0.26	0.99 ± 0.21	4.498	<0.0001^**^
0.01–0.027 Hz	0.81 ± 0.18	0.95 ± 0.16	4.475	<0.0001^**^
0.027–0.073 Hz	0.80 ± 0.16	0.90 ± 0.15	3.607	0.0004^**^
0.073–0.198 Hz	0.78 ± 0.16	0.85 ± 0.15	2.762	0.0065^*^
0.198–0.25 Hz	0.73 ± 0.16	0.76 ± 0.15	1.348	0.1798
**Left superior temporal gyrus**				
0–0.01 Hz	0.89 ± 0.26	0.97 ± 0.27	1.767	0.0794
0.01–0.027 Hz	0.86 ± 0.20	0.90 ± 0.19	1.161	0.2477
0.027–0.073 Hz	0.86 ± 0.18	0.86 ± 0.14	0.126	0.8997
0.073–0.198 Hz	0.95 ± 0.17	0.91 ± 0.15	1.420	0.1577
0.198–0.25 Hz	1.06 ± 0.20	0.98 ± 0.19	2.308	0.0224^*^
**Left white matter**				
0–0.01 Hz	0.75 ± 0.19	0.63 ± 0.19	3.601	0.0004^**^
0.01–0.027 Hz	0.69 ± 0.18	0.62 ± 0.14	2.413	0.0171^*^
0.027–0.073 Hz	0.68 ± 0.12	0.65 ± 0.10	1.549	0.1238
0.073–0.198 Hz	0.69 ± 0.10	0.67 ± 0.08	1.345	0.1807
0.198–0.25 Hz	0.75 ± 0.13	0.76 ± 0.10	0.474	0.6365

*ACC, anterior cingulate gyrus; SMA, supplementary motor cortex*.

*The peak regional homogeneity (ReHo) value is expressed as mean ± SD*.

* *p < 0.05*;

** *p < 0.05/35, 0.001 (Bonferroni correction)*.

The two-sample *t*-tests showed significantly increased ReHo in full bands of the left precentral gyrus and significantly decreased ReHo in full bands of the right operculum (frontal and temporal lobes) (Table 4).

**Table 4:** Two sample *t*-tests for the peak ReHo value of the clusters showed significant differences in ANOVA (group main effect).

**Frequency band**	**TS (*n* = 79)**	**HC (*n* = 63)**	***T*-value**	***P*-value**
	**Mean ± SD**	**Mean ± SD**		
**Left precentral gyrus**				
0–0.01 Hz	0.86 ± 0.25	0.74 ± 0.22	3.521	0.0006^**^
0.01–0.027 Hz	0.87 ± 0.21	0.73 ± 0.18	4.292	0.0003^**^
0.027–0.073 Hz	0.87 ± 0.20	0.75 ± 0.14	4.137	0.00006^**^
0.073–0.198 Hz	0.95 ± 0.19	0.84 ± 0.16	3.590	0.0005^**^
0.198–0.25 Hz	1.03 ± 0.20	0.94 ± 0.17	2.817	0.006^*^
**Right operculum (frontal and temporal lobe)**				
0–0.01 Hz	0.60 ± 0.19	0.73 ± 0.22	−3.632	0.0004^**^
0.01–0.027 Hz	0.61 ± 0.16	0.71 ± 0.18	−3.469	0.0007^**^
0.027–0.073 Hz	0.59 ± 0.15	0.68 ± 0.16	−3.469	0.0007^**^
0.073–0.198 Hz	0.59 ± 0.14	0.66 ± 0.16	−3.040	0.003^*^
0.198–0.25 Hz	0.55 ± 0.12	0.61 ± 0.14	−2.469	0.015^*^

* *p < 0.05*;

** *Bonferroni correction, i.e., p < 0.05/35, 0.001*.

**Figure 4:**
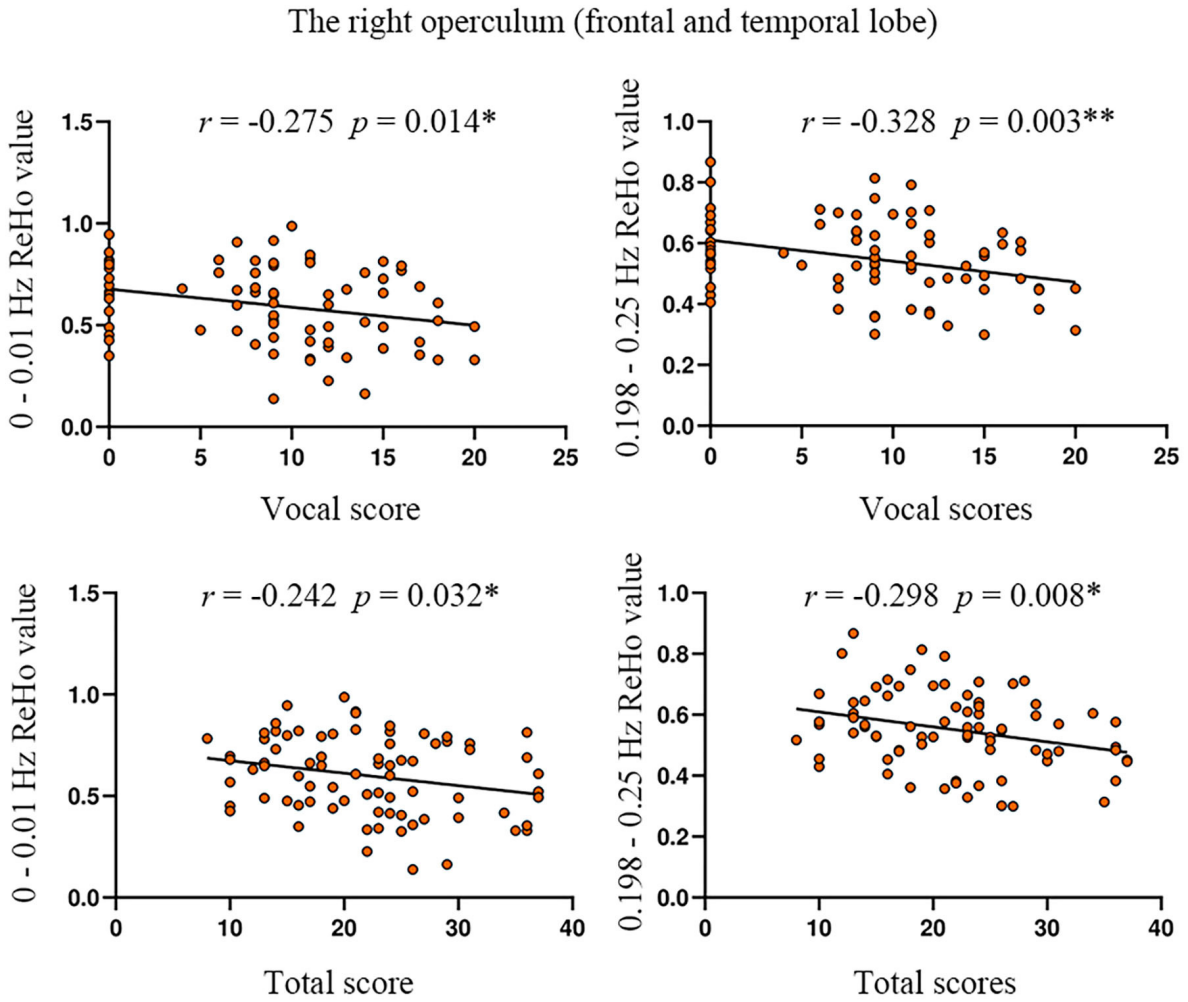
The correlation between ReHo value and YGTSS scores in the right operculum. YGTSS, Yale Global Tic Severity Scale. The total score is the sum of motor score and vocal score. *p < 0.05; **Bonferroni correction, i.e., 0.05/15 = 0.0033.

The ReHo value of the right operculum (frontal and temporal lobes) showed significant negative correlations with vocal scores of YGTSS in the highest frequency bands (0.198–0.25 Hz) (Bonferroni correction, 0.05/15 = 0.0033; Figure 4, Table 5). No significant correlation was found between the ReHo value and any rating score in the left precentral gyrus.

**Table 5:** The correlation between ReHo and the scores of YGTSS in the significantly different brain regions of ANOVA (group main effect).

**Brain area**	**Frequency band**	**ReHo value** **(Mean ± SD)**	**YGTSS score** **(Mean ± SD)**	***R*-value**	***P*-value**
Right operculum (frontal and temporal lobe)	0–0.01 Hz	0.60 ± 0.19	8.67 ± 5.94 (vocal score)	−0.275	0.014^*^
			22.00 ± 7.58 (total score)	−0.242	0.032^*^
	0.198–0.25 Hz	0.55 ± 0.12	8.67 ± 5.94 (vocal score)	−0.328	0.003^**^
			22.00 ± 7.58 (total score)	−0.298	0.008^*^

*The total score is the sum of motor score and vocal score*.

* *p < 0.05*;

** *Bonferroni correction, i.e., p < 0.05/15, 0.0033*.

### Differences of Connectivity Within Networks

**Figure 5:**
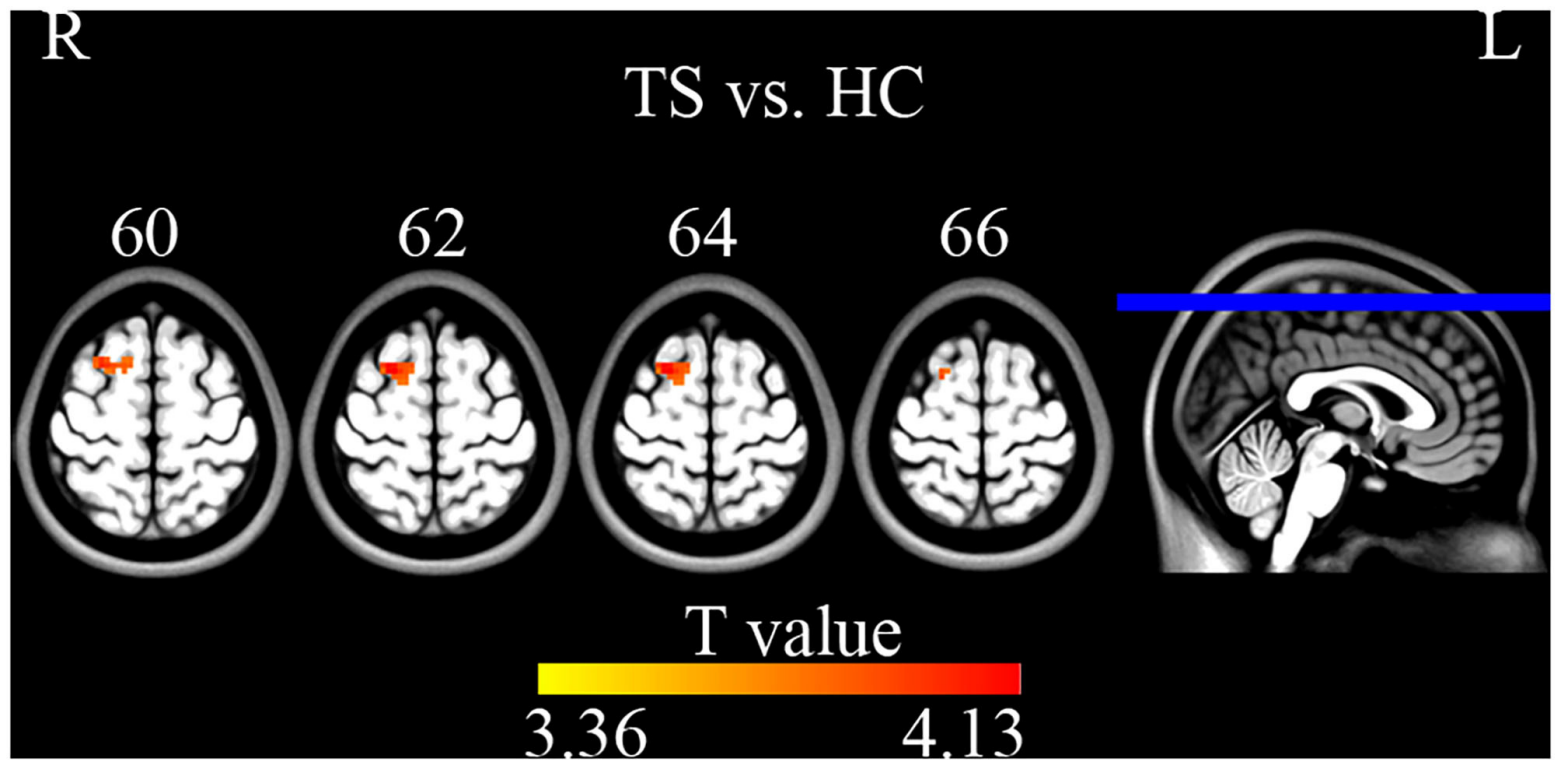
Two sample *t*-tests on ICA maps (GRF correction, single voxel *p* < 0.001, cluster level *p* < 0.05). TS patients showed increased connectivity of the right superior frontal gyrus within the left executive control network.

**Figure 6:**
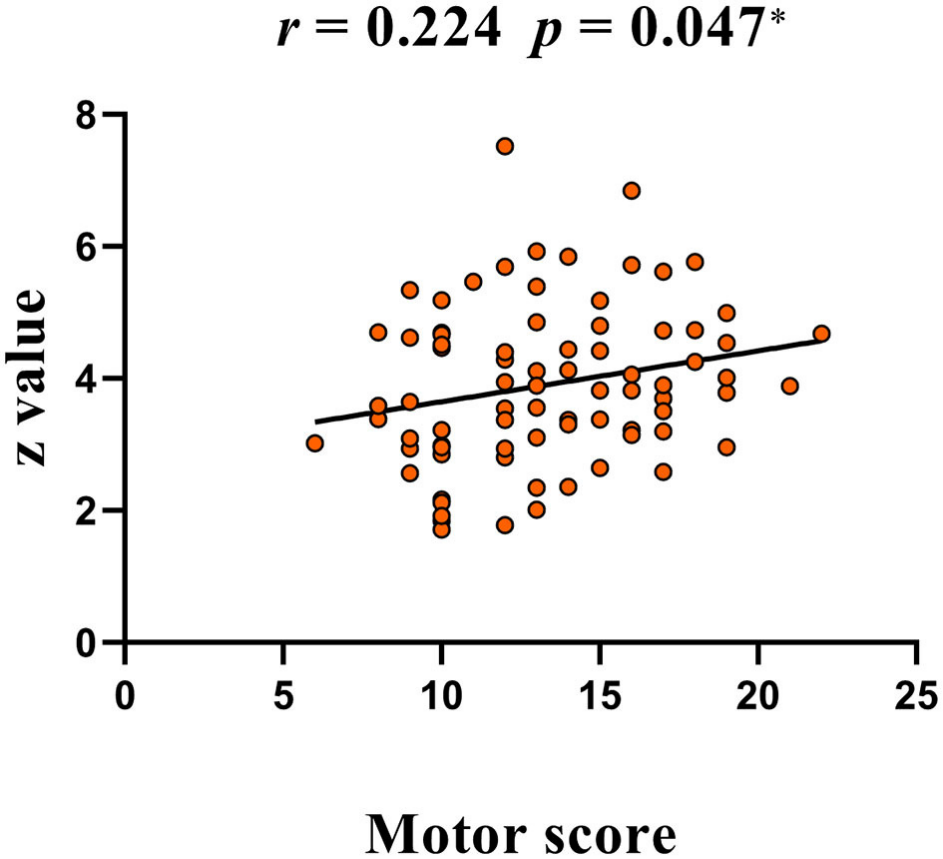
A positive correlation between peak *Z*-value of the right superior frontal gyrus and YGTSS scores (motor score). YGTSS, Yale Global Tic Severity Scale.

Compared with HC, TS patients showed increased connectivity of the right superior frontal gyrus (BA 6, MNI coordinates *X* = 21, *Y* = 9, *Z* = 63, *T* = 4.132, Cluster size = 945 mm^3^) within the left executive control network (Figure 5). No significant group difference was found in the basal ganglia, sensorimotor, and right executive control network. The peak *Z*-value of the right superior frontal gyrus was extracted to perform correlation analysis against clinical assessment scores. Only the motor score of YGTSS showed a positive correlation with abnormal connectivity (Figure 6).

## Discussion

### Reduced Synchronization Within the CSTC Circuit in TS

The present study found decreased ReHo in the right putamen and increased ReHo in the left precentral gyrus of TS children. The putamen, a key node of the basal ganglia, is involved in motor loop circuits (19). The precentral gyrus responds to visual input and codes information during movement preparation (20), which involves cognitive processing for movements such as motor preparation and execution (21). The putamen and precentral gyrus are both parts of the CSTC circuit.

Functional deficits in the CSTC circuit may contribute to the motor symptoms of TS (7). The putamen, the center of the motor portion of the CSTC in the genesis of tics, receives abundant projections from the motor and somatosensory cortices (22). Defects in the modulation of activity in the structures of the motor CSTC network may underlie the failure to inhibit unwanted impulses in patients with TS (7). Previous studies demonstrated the aberrant structure, metabolism, and activity of the putamen and primary motor cortices in TS patients (23–25). Microstructural changes in the white matter of the left precentral gyrus and putamen in unmedicated and pure TS, and the regional apparent diffusion coefficient values were positively correlated with tic severity (25). A proton magnetic resonance spectroscopy study of children with TS showed significant reductions of N-acetylaspartate and choline in the putamen and reduced N-acetylaspartate in the frontal cortical (24). TS children were shown to have increased spontaneous local brain activity in the putamen and bilateral thalamus during RS-fMRI scans (26). Two event-related functional neuroimaging studies support the idea that the primary motor cortex processes preparatory signals related to motor tic behavior in TS (23, 27). One study showed significant fMRI activity in the primary motor cortex before tic onset (23). Another study found that cortical structures, including the primary motor cortex, preceded subcortical activation prior to tic onset (27). Decreased ReHo in the putamen and increased ReHo in the precentral gyrus in the current study indicated reduced synchronization of spontaneous neural activity in the putamen might inhibit the output nuclei of the basal ganglia, which in turn excite the precentral cortex to produce tics.

### Abnormal Synchronization Within Executive Control Networks in TS

Executive control networks are thought to include the dorsolateral prefrontal, anterior cingulate cortices, and the orbitofrontal cortex (28). Many studies provided evidence of possible executive deficits in TS during various executive tasks such as working memory, fluency, shifting, and especially inhibition (29). TS patients could not suppress unwanted movements, and mainly comorbid with ADHD (3). The present study found that ReHo was decreased in the frontal and temporal operculum and ACC, but increased in the superior frontal and parietal cortexes. These regions were reported to be involved in two principal attention control networks. The cingulo-opercular network showed sustained activation across all tasks or nearly all tasks and was hypothesized to maintain task sets (30). The fronto-parietal network, which flexibly segregated or integrated in different aspects of control such as focused proactive control and episodic memory (31), has a crucial role in rapidly adaptive online control (32).

The present results showed reduced synchronization within the cingulo-opercular network (frontal operculum and ACC). Task-maintenance processes may be affected resulting in unwanted breakthroughs of normally suppressed behaviors, such as tics. However, the hyperactive frontal-parietal network (superior frontal and parietal cortexes) motivated initiate and adjust change in task control, again leading to unwanted movements and vocalizations (9). In previous fMRI studies of TS, activation of the operculum, ACC, insular, SMA, primary motor, and somatosensory cortex were found prior to tic onset (23), while decreased amplitude of low-frequency fluctuation (ALFF) and fractional ALFF in the ACC, frontal and parietal gyrus were detected during the resting state (26). In addition, fewer functional connections were found in the fronto-parietal and cingulo-opercular networks in TS children vs. adults and adolescents (9). Abnormalities of metabolism, anatomy, and function in the ACC, an important component of the cingulo-opercular network, have been reported in many previous TS studies (33–37). For instance, significantly decreased gray matter volumes were found in the ACC of TS patients (36). A single-photon emission tomography study reported the hypoperfusion of the ACC during the resting state, which was related to the severity of tics (35), while another study found increased ACC activation during voluntary tic suppression (34, 37). A recent proton magnetic resonance spectroscopy study of TS patients also found that concentrations of glutamine, an important excitatory neurotransmitter, negatively correlated with tic severity scores in ACC (33). Furthermore, we observed a negative correlation between tic severity and ReHoin the operculum. The greater the reduction of synchronization in the operculum, the more serious the disease is thought to become. Above all, altered ReHo of the cingulo-opercular and fronto-parietal networks, especially in the operculum and ACC in the current study, suggested that these brain areas may have an important role in the progress of tic generation mainly by breaking task set maintenance and enhancing adaptive tasks.

In addition to local synchronization, the present study used ICA to detect connectivity differences within the basal ganglia, sensorimotor, and executive control networks. We found increased connectivity of the right superior frontal gyrus within the left executive control network. The executive control network shares major nodes with the frontal-parietal network, including the dorsolateral prefrontal cortex and posterior parietal cortex. It is involved in the control of higher-order cognitive neural functions such as attention, planning, decision-making, and working memory (38). Recent years transcranial magnetic stimulation (TMS) studies attempted to indirectly impact deep or remote brain areas via functional connectivity. Some researchers have successfully observed TMS-induced functional connectivity alterations by stimulating the superficial cortex (39, 40). Our previous work also found increased ReHo in cerebellum after high frequency TMS on precentral gyrus (41). In addition, there was a significant positive correlation between functional connectivity of the right superior frontal gyrus and YGTSS motor score. The right superior frontal gyrus might be a direct or indirect target of TMS for TS treatment. This also suggested that Tics may be aggravated by enhanced functional connectivity within the executive control network, supporting the putative role of the control network in the pathogenesis of TS.

### Frequency-Specific Altered Synchronization in TS

To the best of our knowledge, this is the first study to examine abnormalities of regional synchronization in a relatively large and typical pediatric TS population using the frequency specificity of ReHo. Several studies demonstrated the frequency specificity of ReHo changes in neurologic and psychiatric disorders, such as paroxysmal kinesigenic dyskinesia (42), schizophrenia (43), and major depression (44). In the present study, significantly increased ReHoin the superior frontal gyrus and superior parietal gyrus of TS children indicated compensatory functions of the frontal–parietal network in the lower frequency bands. These findings are consistent with previous reports that the lower frequency band (0.01–0.027 Hz) had a higher BOLD fluctuation in the cortical regions (13). We also found decreased ReHo in the putamen, ACC, and superior temporal gyrus in the lower-frequency bands, which was normal or reversed in the higher frequency bands. Previous RS-fMRI studies indicated that the higher band (0.027–0.073 Hz) exhibited increased ALFF compared with the lower band (0.01–0.027 Hz) in the subcortical regions (13, 45). The interaction of frequency band × group on ReHo indicated a significant difference in frequency-specific alterations (Figure 3, Table 3). The results of the current study suggested that the local intrinsic brain activity of TS was sensitive to specific frequency bands. More frequency-specific investigations are needed in the future.

The significant negative correlation between ReHo and YGTSS scores was found only in the highest frequency bands (0.198–0.25 Hz) (Figure 4, Table 5), suggesting that the ultra-high frequency band might be of clinical importance. These findings indicate that abnormal ReHoin TS is frequency-dependent and might be missed when using routine frequency band. Although the nature of these frequency-specific alterations of local neuronal homogeneity is still unclear, different frequency bands should be considered when measuring the ReHo of TS children in future studies to further understand the pathology of TS.

## Conclusion

In summary, the present RS-fMRI study revealed frequency-specific abnormal regional homogeneity and altered connectivity in children with TS. The regions of frequency-specific abnormalities included the frontal–parietal network, ACC, and putamen, as well as an altered connectivity region in the executive control network. These brain regions are involved in multiple cognitive dysfunctions characteristic of TS. All these aberrant regions might be direct treatment targets or indirect targets for brain stimulation, especially those regions with frequency-specific abnormalities, which might be sensitive to specific stimulation frequencies for TMS. These targets should be tested and verified in TS populations in the future.

## Limitations

This study had several limitations. The drugs used for TS treatment may affect brain function. The dose and duration of drug information were not recorded completely in the current study. The variability of the doses and types of drugs used makes it difficult to analyze the contribution of each drug on ReHo. Therefore, drug-naïve status and type of medication used should be taken into consideration in the future.

## Data Availability Statement

All datasets generated for this study are included in the article/supplementary material.

## Ethics Statement

The studies involving human participants were reviewed and approved by Medical Ethics Committee of the Center for Cognition and Brain Disorders, Hangzhou Normal University, China. Written informed consent to participate in this study was provided by the participants' legal guardian/next of kin. Written informed consent was obtained from the individual(s), and minor(s)' legal guardian/next of kin, for the publication of any potentially identifiable images or data included in this article.

## Author Contributions

Y-TL: design the study, statistical analysis, and writing of the first draft. X-LL: fMRI data processing and statistical analysis. YW: neurological evaluation and psychiatric evaluation. G-JJ: fMRI data acquisition. Y-FZ: revised the article and developed the research concept. JW: fMRI data processing and statistical analysis, revised the article, and developed the research concept. J-HF: research project conception and organization, neurological evaluation, revised the article, and developed the research concept. All authors: contributed to the article and approved the submitted version.

## Conflict of Interest

The authors declare that the research was conducted in the absence of any commercial or financial relationships that could be construed as a potential conflict of interest.

## References

[1] Swain JE Scahill L Lombroso PJ King RA Leckman JF. Tourette syndrome and tic disorders: a decade of progress. J Am Acad Child Adolesc Psychiatry. (2007) 46:947–68. 10.1097/chi.0b013e318068fbcc 17667475

[2] Yang C Zhang L Zhu P Zhu C Guo Q. The prevalence of tic disorders for children in China: a systematic review and meta-analysis. Medicine. (2016) 95:e4354. 10.1097/MD.0000000000004354 27472724 PMC5265861

[3] Mol Debes NM Hjalgrim H Skov L Validation of the presence of comorbidities in a Danish clinical cohort of children with tourette syndrome. J Child Neurol. (2008) 23:1017–27. 10.1177/0883073808316370 18827268

[4] Evans J Seri S Cavanna AE. The effects of gilles de la tourette syndrome and other chronic tic disorders on quality of life across the lifespan: a systematic review. Eur Child Adolesc Psychiatry. (2016) 25:939–48. 10.1007/s00787-016-0823-8 26880181 PMC4990617

[5] Debes NM Skov L Hjalgrim H. [Tourette syndrome. Genetics, neuroanatomy and neurotransmitters]. Ugeskr Laeger. (2008) 170:2695–700. 18761860

[6] Greene DJ Schlaggar BL Black KJ. Neuroimaging in tourette syndrome: research highlights from 2014–2015. Curr Dev Disord Rep. (2015) 2:300–8. 10.1007/s40474-015-0062-6 26543796 PMC4629775

[7] Mink JW. Neurobiology of basal ganglia and tourette syndrome: basal ganglia circuits and thalamocortical outputs. Adv Neurol. (2006) 99:89–98. 16536354

[8] Polyanska L Critchley HD Rae CL. Centrality of prefrontal and motor preparation cortices to Tourette syndrome revealed by meta-analysis of task-based neuroimaging studies. Neuroimage Clin. (2017) 16:257–67. 10.1016/j.nicl.2017.08.004 28831377 PMC5554925

[9] Church JA Fair DA Dosenbach NU Cohen AL Miezin FM Petersen SE. Control networks in paediatric tourette syndrome show immature and anomalous patterns of functional connectivity. Brain. (2009) 132:225–38. 10.1093/brain/awn223 18952678 PMC2638693

[10] Ganos C Kahl U Brandt V Schunke O Baumer T Thomalla G. The neural correlates of tic inhibition in gilles de la tourette syndrome. Neuropsychologia. (2014) 65:297–301. 10.1016/j.neuropsychologia.2014.08.007 25128587

[11] Liu Y Wang J Zhang J Wen H Zhang Y Kang H. Altered spontaneous brain activity in children with early Tourette syndrome: a resting-state fMRI study. Sci Rep. (2017) 7:4808. 10.1038/s41598-017-04148-z 28684794 PMC5500479

[12] Wu CW Gu H Lu H Stein EA Chen JH Yang Y. Frequency specificity of functional connectivity in brain networks. Neuroimage. (2008) 42:1047–55. 10.1016/j.neuroimage.2008.05.035 18632288 PMC2612530

[13] Zuo XN Di Martino A Kelly C Shehzad ZE Gee DG Klein DF. The oscillating brain: complex and reliable. Neuroimage. (2010) 49:1432–45. 10.1016/j.neuroimage.2009.09.037 19782143 PMC2856476

[14] Song X Zhang Y Liu Y. Frequency specificity of regional homogeneity in the resting-state human brain. PLoS ONE. (2014) 9:e86818. 10.1371/journal.pone.0086818 24466256 PMC3900644

[15] Wang J Zhang Z Ji GJ Xu Q Huang Y Wang Z. Frequency-specific alterations of local synchronization in idiopathic generalized epilepsy. Medicine. (2015) 94:e1374. 10.1097/MD.0000000000001374 26266394 PMC4616718

[16] Yan CG Wang XD Zuo XN Zang YF. DPABI: data processing & analysis for (resting-state) brain imaging. Neuroinformatics. (2016) 14:339–51. 10.1007/s12021-016-9299-4 27075850

[17] Yan CG Cheung B Kelly C Colcombe S Craddock RC Di Martino A. A comprehensive assessment of regional variation in the impact of head micromovements on functional connectomics. Neuroimage. (2013) 76:183–201. 10.1016/j.neuroimage.2013.03.004 23499792 PMC3896129

[18] Li YO Adali T Calhoun VD. Estimating the number of independent components for functional magnetic resonance imaging data. Hum Brain Mapp. (2007) 28:1251–66. 10.1002/hbm.20359 17274023 PMC6871474

[19] Middleton FA Strick PL. Basal ganglia and cerebellar loops: motor and cognitive circuits. Brain Res Brain Res Rev. (2000) 31:236–50. 10.1016/S0165-0173(99)00040-5 10719151

[20] Gallivan JP McLean DA Flanagan JR Culham JC. Where one hand meets the other: limb-specific and action-dependent movement plans decoded from preparatory signals in single human frontoparietal brain areas. J Neurosci. (2013) 33:1991–2008. 10.1523/JNEUROSCI.0541-12.2013 23365237 PMC6619126

[21] Smith FW Goodale MA. Decoding visual object categories in early somatosensory cortex. Cereb Cortex. (2015) 25:1020–31. 10.1093/cercor/bht292 24122136 PMC4380001

[22] Alexander GE DeLong MR Strick PL. Parallel organization of functionally segregated circuits linking basal ganglia and cortex. Annu Rev Neurosci. (1986) 9:357–81. 10.1146/annurev.ne.09.030186.002041 3085570

[23] Bohlhalter S Goldfine A Matteson S Garraux G Hanakawa T Kansaku K. Neural correlates of tic generation in tourette syndrome: an event-related functional MRI study. Brain. (2006) 129:2029–37. 10.1093/brain/awl050 16520330

[24] DeVito TJ Drost DJ Pavlosky W Neufeld RW Rajakumar N McKinlay BD. Brain magnetic resonance spectroscopy in tourette's disorder. J Am Acad Child Adolesc Psychiatry. (2005) 44:1301–8. 10.1097/01.chi.0000181046.52078.f4 16292123

[25] Muller-Vahl KR Grosskreutz J Prell T Kaufmann J Bodammer N Peschel T. Tics are caused by alterations in prefrontal areas, thalamus and putamen, while changes in the cingulate gyrus reflect secondary compensatory mechanisms. BMC Neurosci. (2014) 15:6. 10.1186/1471-2202-15-6 24397347 PMC3893393

[26] Cui Y Jin Z Chen X He Y Liang X Zheng Y. Abnormal baseline brain activity in drug-naive patients with tourette syndrome: a resting-state fMRI study. Front Hum Neurosci. (2014) 7:913. 10.3389/fnhum.2013.00913 24427134 PMC3877773

[27] Neuner I Werner CJ Arrubla J Stocker T Ehlen C Wegener HP. Imaging the where and when of tic generation and resting state networks in adult tourette patients. Front Hum Neurosci. (2014) 8:362. 10.3389/fnhum.2014.00362 24904391 PMC4035756

[28] Zgaljardic DJ Borod JC Foldi NS Mattis PJ Gordon MF Feigin A. An examination of executive dysfunction associated with frontostriatal circuitry in Parkinson's disease. J Clin Exp Neuropsychol. (2006) 28:1127–44. 10.1080/13803390500246910 16840240 PMC4456005

[29] Eddy CM Rizzo R Cavanna AE. Neuropsychological aspects of tourette syndrome: a review. J Psychosom Res. (2009) 67:503–13. 10.1016/j.jpsychores.2009.08.001 19913655

[30] Dosenbach NU Visscher KM Palmer ED Miezin FM Wenger KK Kang HC. A core system for the implementation of task sets. Neuron. (2006) 50:799–812. 10.1016/j.neuron.2006.04.031 16731517 PMC3621133

[31] Ray KL Ragland JD MacDonald AW Gold JM Silverstein SM Barch DM. Dynamic reorganization of the frontal parietal network during cognitive control and episodic memory. Cogn Affect Behav Neurosci. (2020) 20:76–90. 10.3758/s13415-019-00753-9 31811557 PMC7018593

[32] Dosenbach NU Fair DA Cohen AL Schlaggar BL Petersen SE. A dual-networks architecture of top-down control. Trends Cogn Sci. (2008) 12:99–105. 10.1016/j.tics.2008.01.001 18262825 PMC3632449

[33] Fan S Cath DC van den Heuvel OA van der Werf YD Schols C Veltman DJ. Abnormalities in metabolite concentrations in tourette's disorder and obsessive-compulsive disorder-A proton magnetic resonance spectroscopy study. Psychoneuroendocrinology. (2017) 77:211–17. 10.1016/j.psyneuen.2016.12.007 28104554

[34] Kawohl W Bruhl A Krowatschek G Ketteler D Herwig U. Functional magnetic resonance imaging of tics and tic suppression in gilles de la tourette syndrome. World J Biol Psychiatry. (2009) 10:567–70. 10.1080/15622970802118356 18609432

[35] Moriarty J Costa DC Schmitz B Trimble MR Ell PJ Robertson MM. Brain perfusion abnormalities in gilles de la tourette's syndrome. Br J Psychiatry. (1995) 167:249–54. 10.1192/bjp.167.2.249 7582678

[36] Muller-Vahl KR Kaufmann J Grosskreutz J Dengler R Emrich HM Peschel T. Prefrontal and anterior cingulate cortex abnormalities in tourette syndrome: evidence from voxel-based morphometry and magnetization transfer imaging. BMC Neurosci. (2009) 10:47. 10.1186/1471-2202-10-47 19435502 PMC2691409

[37] van der Salm SMA van der Meer JN Cath DC Groot PFC van der Werf YD Brouwers E. Distinctive tics suppression network in Gilles de la Tourette syndrome distinguished from suppression of natural urges using multimodal imaging. Neuroimage Clin. (2018) 20:783–92. 10.1016/j.nicl.2018.09.014 30268027 PMC6169325

[38] Menon V. Large-scale brain networks and psychopathology: a unifying triple network model. Trends Cogn Sci. (2011) 15:483–506. 10.1016/j.tics.2011.08.003 21908230

[39] Eldaief MC Halko MA Buckner RL Pascual-Leone A. Transcranial magnetic stimulation modulates the brain's intrinsic activity in a frequency-dependent manner. Proc Natl Acad Sci USA. (2011) 108:21229–34. 10.1073/pnas.1113103109 22160708 PMC3248528

[40] Wang JX Rogers LM Gross EZ Ryals AJ Dokucu ME Brandstatt KL. Targeted enhancement of cortical-hippocampal brain networks and associative memory. Science. (2014) 345:1054–7. 10.1126/science.1252900 25170153 PMC4307924

[41] Wang J Deng XP Wu YY Li XL Feng ZJ Wang HX. High-frequency rTMS of the motor cortex modulates cerebellar and widespread activity as revealed by SVM. Front. Neurosci. (2020) 14:186. 10.3389/fnins.2020.00186 32265624 PMC7096733

[42] Liu ZR Miao HH Yu Y Ding MP Liao W. Frequency-specific local synchronization changes in paroxysmal kinesigenic dyskinesia. Medicine. (2016) 95:e3293. 10.1097/MD.0000000000003293 27043701 PMC4998562

[43] Yu R Hsieh MH Wang HL Liu CM Liu CC Hwang TJ. Frequency dependent alterations in regional homogeneity of baseline brain activity in schizophrenia. PLoS ONE. (2013) 8:e57516. 10.1371/journal.pone.0057516 23483911 PMC3590274

[44] Xue S Wang X Wang W Liu J Qiu J. Frequency-dependent alterations in regional homogeneity in major depression. Behav Brain Res. (2016) 306:13–9. 10.1016/j.bbr.2016.03.012 26968135

[45] Gao L Bai L Zhang Y Dai XJ Netra R Min Y. Frequency-dependent changes of local resting oscillations in sleep-deprived brain. PLoS ONE. (2015) 10:e0120323. 10.1371/journal.pone.0120323 25798918 PMC4370559

